# Remote ischemic conditioning for acute respiratory distress syndrome in COVID-19

**DOI:** 10.1152/ajplung.00223.2020

**Published:** 2021-01-06

**Authors:** Anthony V. Incognito, Philip J. Millar, W. Glen Pyle

**Affiliations:** ^1^Department of Human Health and Nutritional Sciences, College of Biological Sciences, University of Guelph, Guelph, Ontario, Canada; ^2^Department of Physiology and Pharmacology, Cumming School of Medicine, University of Calgary, Calgary, Alberta, Canada; ^3^Toronto General Research Institute, Toronto, Ontario, Canada; ^4^Department of Biomedical Sciences, Ontario Veterinary College, University of Guelph, Guelph, Ontario, Canada; ^5^IMPART Team Canada Investigator Network, Dalhousie Medicine, Dalhousie University, Saint John, New Brunswick, Canada

**Keywords:** acute respiratory distress syndrome, COVID-19, remote ischemic conditioning

## Abstract

Acute respiratory distress syndrome and subsequent respiratory failure remains the leading cause of death (>80%) in patients severely impacted by COVID-19. The lack of clinically effective therapies for COVID-19 calls for the consideration of novel adjunct therapeutic approaches. Though novel antiviral treatments and vaccination hold promise in control and prevention of early disease, it is noteworthy that in severe cases of COVID-19, addressing “run-away” inflammatory cascades are likely more relevant for improvement of clinical outcomes. Viral loads may decrease in severe, end-stage coronavirus cases, but a systemically damaging cytokine storm persists and mediates multiple organ injury. Remote ischemic conditioning (RIC) of the limbs has shown potential in recent years to protect the lungs and other organs against pathological conditions similar to that observed in COVID-19. We review the efficacy of RIC in protecting the lungs against acute injury and current points of consideration. The beneficial effects of RIC on lung injury along with other related cardiovascular complications are discussed, as are the limitations presented by sex and aging. This adjunct therapy is highly feasible, noninvasive, and proven to be safe in clinical conditions. If proven effective in clinical trials for acute respiratory distress syndrome and COVID-19, application in the clinical setting could be immediately implemented to improve outcomes.

## INTRODUCTION

The global COVID-19 pandemic, announced by the World Health Organization on March 11, 2020, characterizes the clinical manifestation of the severe acute respiratory syndrome coronavirus 2 (SARS-CoV-2) infection. SARS-CoV-2 infection evokes pneumonia, fever, and strong inflammatory responses that can progress to acute respiratory distress syndrome (ARDS) (1–4). It has been reported that >80% of nonsurviving patients suffer from ARDS and/or respiratory failure, often with associated cardiovascular injury (3–6). The lungs of patients with COVID-19 are subject to interstitial mononuclear cell infiltration and edema (7, 8), characteristic outcomes in ARDS (9).

## ARDS IN COVID-19 AND RELATED CONDITIONS

**Figure 1.**
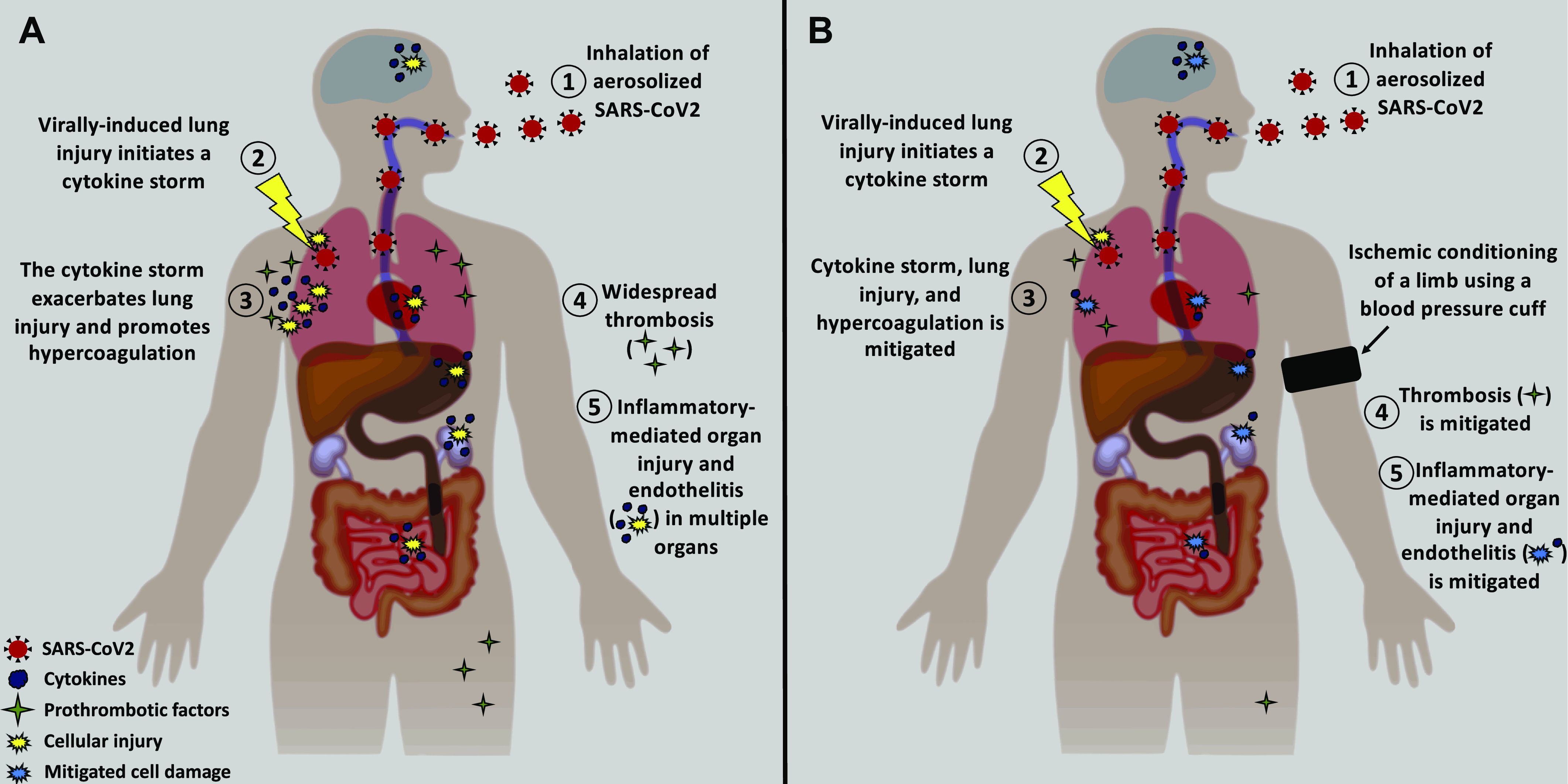
*A*: the pathophysiology of SARS-CoV-2-induced lung and systemic organ injury and thrombosis. After inhalation, the viral infection of lung cells leads to cell death and localized lung injury. Lung cell death initiates a cytokine storm and hypercoagulation through the release of cytokines and prothrombotic factors, locally and systemically. The localized inflammatory response exacerbates the lung injury. The systemic inflammation further promotes a systemic hypercoagulation state and endothelitis, leading to widespread thrombosis and injury to multiple organs. *B*: after the viral infection, the application of ischemic conditioning of a limb can be done by inflating a blood pressure cuff for 5 min, followed by a 5-min reperfusion period, repeated three to four times. This procedure has been shown to mitigate inflammation and inflammation-induced cellular injury and is also thrombolytic. Thus, remote ischemic conditioning may offer protection against the debilitating cascade observed in severe cases of COVID-19 (i.e., stages 3, 4, and 5 in the figure).

Our current understanding is that SARS-CoV-2 infection relies largely on the virus binding to membrane-bound angiotensin converting enzyme-2 (ACE2) and priming by transmembrane serene protease-2 (TMPRSS2) (10). Both proteins are highly populated on transient secretory cells within the lung (11). The lungs represent a primary exposure site for infection given the virus’ ability to be transmitted via aerosolization from respiratory droplets (12). Once infected, cell death can ensue, releasing a plethora of cytokines (e.g., IL-2, IL-6, IL-8, IL-1β, IFN-γ, TNFα) (13–16) into the blood for systemic distribution, effectively initiating a cytokine storm (Fig. 1*A*). Indeed, the levels of circulating cytokines (or cytokine-induced C reactive protein) and leukocytes appear to directly relate to disease severity (2, 4, 8, 17) and are predictive of mortality (4, 6, 17). Subsequent to the cytokine storm, recruitment of circulating leukocytes to the lung occurs, leading to an increase in reactive oxygen species (ROS) production, a decrease in alveolar-capillary barrier integrity, and an increase in the expression of prothrombotic factors, as reviewed previously (18). Together, these processes can exacerbate the acute lung injury [epithelial cell death, local inflammation, endothelial and epithelial barrier impairment, and subsequent lung edema (19)] beyond that directly caused by the viral infection itself and can lead to ARDS, as well as pulmonary embolism in some patients (20). In addition, given the decreased alveolar-capillary barrier integrity, these inflammatory mediators can leak into peripheral circulation and drive widespread thrombosis and endothelitis, sepsis, and multiple organ failure (4, 5, 8, 21–24), which are relevant in ARDS (9).

Surgical procedures requiring prolonged cessation of blood flow to the lungs can cause ischemia-reperfusion (IR) injury and manifest ARDS. For example, lung IR is observed in patients after pulmonary resection/lobectomy procedures, where the lung is collapsed (atelectatic) and hypoperfused for the surgical duration (∼1–2 h), followed by rapid reinflation and reperfusion of the lung tissue. In IR, tissue cell necrosis initiates the release of damage-associated molecular patterns (DAMPs), pro- and anti-inflammatory cytokines, and ROS, as well as recruitment of circulating macrophages and neutrophils that increase local vascular permeability and generate prothrombotic factors (25). The cascade associated with IR injury appears to closely resemble ARDS mechanisms reported in COVID-19 (26). Noteworthy is that IR of any organ can initiate a systemic inflammatory response, which can lead to inflammation-mediated injury to distant organs. The systemic inflammatory response triggered by ARDS is currently understood as a mechanism for multiple organ failure in patients with COVID-19 (21, 23).

There has been large focus, mainly in surgical arenas, on developing clinical strategies to ameliorate IR- and inflammation-induced ARDS (27). A promising adjunct therapy for lung protection from surgery-induced infarction and inflammation that has been under in-depth investigation is remote ischemic conditioning (RIC). Given the pathological similarities between surgical IR and ARDS and the pulmonary dysfunction associated with COVID-19, investigating therapeutic strategies that protect against the lung injury induced by surgical procedures may provide unique insights into the management of pneumonia-induced ARDS in COVID-19. This review seeks to explore the potential of using RIC as a viable clinical strategy to minimize the respiratory distress associated with COVID-19.

## REMOTE ISCHEMIC CONDITIONING

RIC is the application of brief, nonlethal bouts of an ischemic challenge before (preconditioning), during (perconditioning), or after (postconditioning) a severe stress in a distant organ (28). Ischemic conditioning was first shown in the canine myocardium, where the infarct size following a 40-min coronary artery occlusion and reperfusion (i.e., IR) was significantly reduced by ∼23% if the occlusion was preceded (i.e., preconditioned) by brief nonlethal ischemic challenges of the same myocardial region (29). Following the discovery of myocardial preconditioning, ischemic conditioning was shown to be cardioprotective against IR even when the nonlethal ischemic challenges were administered in a remote myocardial region (30) and even in organs outside the heart (31). The ability to mediate cardioprotection through the conditioning of distant organs with repetitive, sublethal ischemic episodes served as the foundation of extending RIC investigations to demonstrate efficacy in multiorgan protection (27). Presently, the remote organ protection is understood to be mediated by the release of blood-borne protective factor(s), mediated by a complex, integrative neurohumoral signal transduction cascade requiring an intact sensory innervation to the remote region made ischemic, intact vagal efferent and muscarinic receptor signaling (32), and splenic innervation (33). The intracellular mechanisms of protection mediated by remote ischemic conditioning have yet to be fully elucidated, but there are noted similarities with local ischemic conditioning. Activation of RISK and/or SAFE signaling by humoral factors has been identified as key factors in protecting against myocardial injury, as has PKC and nitric oxide signaling, which have distinct pathways as well as interaction with RISK/SAFE elements (34). Most studies have identified mitochondria as central targets in the intracellular signaling cascade, although work by Gedik et al. (35) suggests that the specific role of mitochondria in mediating protection and the extracellular factors responsible for initiating the intracellular cascade may differ across species.

The first report of the effectiveness of RIC in humans was against IR-induced endothelial dysfunction (36). A manual sphygmomanometer was inflated over the upper arm to suprasystolic pressures (200 mmHg) for 5 min to induce a noninjurious ischemic challenge. This was repeated three times with 5-min reperfusion periods in between bouts. The 25-min protocol was performed before a prolonged (20 min) ischemic insult on the contralateral arm. Endothelial dysfunction induced by the ischemic insult was completely abolished when subjects were remotely preconditioned. In a follow-up investigation, application of ischemic challenges during, rather than before, the ischemic insult (i.e., perconditioning) offered a similar degree of protection against endothelial dysfunction, though only in experiments that applied the ischemic challenges to the leg, not the arm (37). Moreover, two bouts of ischemic challenge before the prolonged ischemic insult in the leg was equally protective as the minimum required three bouts in the arm. These findings suggested that a threshold level of a remote ischemic stimulus, mediated by the number of ischemic challenges and/or the muscle mass subjected to the ischemic challenges was required to elicit cytoprotective effects. Since these discoveries, RIC in humans is most commonly applied to the arm or leg using three or four 5-min bouts of ischemic challenges interspersed with equal periods of reperfusion (28). The noninvasive nature of this technique, the ease of application, and the wide array of successes in protecting against IR in a number of organ systems has garnered much clinical interest (27).

## LUNG PROTECTION WITH REMOTE ISCHEMIC CONDITIONING

There are several animal models whereby RIC has shown promise in protecting against lung injury arising from different stressors (i.e., surgery, IR, systemic inflammatory insults) with similar pathological mechanisms to COVID-19 ARDS. Hindlimb ischemic preconditioning in a porcine model of pulmonary IR was shown to completely protect against impaired gas exchange and pulmonary hypertension, as well as the systemic rise in the levels of macrophages and IL-1β, but not IL-6 or ROS (38). In adult Sprague-Dawley rats, preconditioning of the liver before left lung IR reduced lung injury, edema, cytokine production, and leukocyte recruitment (39). Furthermore, bilateral hindlimb preconditioning in adult Sprague-Dawley rats attenuated acute lung injury and neutrophil infiltration following cardiopulmonary bypass surgery, as well as abolished gas exchange impairment and attenuated reductions in circulating anti-inflammatory cytokines (IL-4 and IL-10) (40).

In a shock model using Sprague-Dawley rats, hemorrhagic shock and resuscitation was used to induce acute lung injury, which coincided with lung inflammation, edema, leukocyte infiltration, and increases in plasma IL-6 (41). Hindlimb ischemic preconditioning partially ameliorated these negative effects. Similar results using a hemorrhagic shock and resuscitation model were also observed in mice, in addition to a complete abolishment of a systemic inflammatory response, though these responses were not observed when ischemic challenges were evoked during (perconditioning) or after (postconditioning) the stressor (42). In addition, this study also observed a reduction in neutrophil migration to the site of injury in zebrafish treated with ischemic conditioned plasma from the mice. Moreover in mice, systemic inflammation could also be evoked by intraperitoneal lipopolysaccharide administration (septic shock model), which induces large increases in cytokine concentrations in bronchoalveolar lavage fluid, as well as acute lung injury and pulmonary edema (43). Hindlimb preconditioning was partially protective against the indirect inflammation-mediated lung injury. Ischemic postconditioning in the septic shock model was also shown to attenuate rises in systemic inflammation and neutrophil accumulation (tested in liver tissue) (44).

Similar promise with remote conditioning has been shown in humans. Remote ischemic preconditioning of the leg improved gas exchange and lowered lung oxidative stress following lung lobectomy (45). Likewise, remote ischemic preconditioning of the arm was also efficacious in reducing impaired gas exchange and the incidence of acute lung injury after pulmonary resection surgery, which coincided with reductions in the systemic inflammatory response (IL-6, TNFα), oxidative stress, and length of postoperative hospital stay (46). In cardiopulmonary bypass surgery, pairing pre- and postconditioning of the arm increased gas exchange postsurgery and reduced the incidence of patients requiring >48 h ventilation time (47). Preconditioning alone before cardiopulmonary bypass surgery was also shown to reduce systemic neutrophil counts (48). Similarly, perconditioning of the leg during valve replacement surgery decreased incidence of acute lung injury, though the systemic inflammatory response was unchanged (49). Last, an ongoing clinical trial is testing the effects of upper limb ischemic perconditioning in ICU patients with septic shock on multiple organ failure scores, which includes a measure of pulmonary gas exchange (50).

Though these models of IR and inflammation-mediated lung injury are similar to the pathophysiology of COVID-19, no studies have directly assessed the efficacy of RIC on pneumonia-induced lung injury. Indirect evidence in patients with obstructive sleep apnea provides insight into the promising potential of using RIC to protect against COVID-19 ARDS. Patients with obstructive sleep apnea undergo systemic intermittent hypercapnic hypoxia from repeat apneas. Though intermittent hypoxia is different from limb ischemic conditioning, similar protective mechanisms may be manifested systemically, as both stimuli have been shown to induce similar protective cellular signaling pathways mediated by reactive oxygen species, hypoxia-inducible factor 1, protein kinase C, nitric oxide, antioxidant enzymes, and the mitochondrial K-ATP channel, via common triggers such as adenosine, adrenergic, and opioid receptor activation (51–53). Interestingly, there is a lowered mortality and increase in nonroutine discharge rates in mechanically ventilated patients with community-acquired pneumonia that have underlying obstructive sleep apnea (54). Patients with obstructive sleep apnea with myocardial infarction also demonstrate reduced myocardial injury (55). Indeed, preconditioning with intermittent periods of ceased ventilation of the operable, nondependent lung before pulmonary lobectomy surgery has been shown to reduce lung injury and inflammation assessed postsurgery (56), which has also been observed with RIC in similar clinical settings (45, 46). Although there is a plethora of comorbid conditions associated with obstructive sleep apnea (including an increased incidence of myocardial infarction (57)) which appears dose-dependent (i.e., number of hypoxic hypercapnic episodes per day), the condition could possess mechanisms beneficial toward clinical outcomes in pneumonia, as well as for infarction size in the setting of myocardial infarction, suggesting the possibility that harnessing the protective mechanisms associated with this condition, potentially with the controlled use of RIC, may be beneficial in COVID-19.

## PROTECTION AGAINST COVID-19-RELATED CARDIOVASCULAR COMPLICATIONS WITH REMOTE ISCHEMIC CONDITIONING

The initial reports of COVID-19 identified the condition as a respiratory illness. Although it is true that significant clinical symptoms, including respiratory distress, and the use of the respiratory system as a means of entry, support a critical role for the lungs in the pathogenesis of COVID-19, emerging and established evidence points to a more systemic disease. Arguably the most widely affected system besides the lungs is the cardiovascular system. Severely ill patients experience multiorgan endothelitis and other vascular-based disorders, consequent of the systemic propagation of the cytokine storm from lung infection, as well as from direct endothelial viral infection (24). Furthermore, myocardial injury is a hallmark of severe disease (5). Although the widespread impact of SARS-CoV-2 infection is concerning and presents a profound clinical challenge, RIC is an intervention that represents a systemic therapeutic strategy whose effects may provide benefit and protection to a diverse range of organ and tissue systems impacted by COVID-19.

An emerging challenge in the clinical management of patients with COVID-19 is the development of widespread thrombosis. Patients with COVID-19 exhibit elevated markers of hypercoagulation that correspond with negative outcomes, and deep vein thrombosis and pulmonary embolism are reported at unusually high rates (20). Equally concerning is the fact that some medications that are under investigation may exacerbate the risk of thrombosis. The antiviral therapies lopinavir/ritonavir and remdesivir interfere with common blood-clotting medications (20). Furthermore, lopinavir/ritonavir alters serum lipids to increase the risk of atherosclerosis (58), which may exacerbate thrombotic events. However, recent reports of arrhythmogenic effects from lopinavir/ritonavir (59, 60) and remdesivir (61) as well as other adverse side effects (62) may prevent further application in COVID-19 clinical settings. RIC accelerates thrombolysis and decreases platelet-mediated thrombosis in a range of animal models and human patients, while proving safe in a variety of clinical settings (63, 64), although the mechanisms underlying the protective effects are not yet determined (65). Furthermore, endothelial cell function is maintained by remote conditioning after a pathological stressor, which helps to preserve the vascular barrier and maintain its antithrombotic potential (27, 28). The prothrombotic threats seen in COVID-19 patients with ARDS offer the potential for RIC to affect protective benefits by reducing the risk of blood clots, including pulmonary embolism. However, to date the pathological stressors studied in RIC have largely been confined to ischemia, and thus generalizability to COVID-19 should be cautioned, though warranting of clinical investigation.

In addition to the blood clotting disorders that characterize SARS-CoV-2 infection, impairment to the microcirculation has been reported in patients with COVID-19 (5). Vascular dysfunction has the potential to impact virtually every system in the body, and mitigation of vascular injury could conceivably improve patient outcomes. It has been reported that local and RIC protects the heart by not only decreasing microemboli but also reducing vascular complications, including increased vascular permeability, endothelial dysfunction, and capillary destruction (28). Also apparent is the clinical viability of RIC of surgical flaps in plastic surgery, whereby the microcirculation of surgical flaps is protected with ischemic preconditioning (66). The positive impact of RIC on the vasculature extends the potential benefits of RIC in patients with COVID-19 by mitigating one of the most significant injuries associated with SARS-CoV-2 infection.

## SEX DIFFERENCES IN DISEASE AND TREATMENT

The prevalence of COVID-19 is generally comparable between men and women across the globe (67). However, men tend to experience more severe illness, evident by the greater representation and more severe complications of those hospitalized, which drives a mortality rate that is ∼2 times higher than that of women (67). The biological reason for these disease severity differences is not known, although it is widely recognized that men have higher rates of comorbidities, such as cardiovascular and respiratory diseases (67), which are linked to worse outcomes (3–5), as well as the propensity for men to develop ARDS and respiratory failure (1, 4, 68).

Sex-specific treatment strategies have emerged as an important consideration in the clinical management of disease. Sex differences in the response to RIC are inconsistent and not widely studied, which makes it difficult to determine if this treatment would equally benefit males and females. In a recent investigation, isolated male rat hearts (Langendorff) were treated with the plasma of young men and women before and after ischemic conditioning of the arm (69), which is the transport medium of the cytoprotective humoral factor(s) after ischemic conditioning (27, 28). The infarct size following IR was significantly reduced by pretreatment with ischemic conditioned plasma; however, this was only observed using plasma collected from men. Whether this is due to the ischemic challenges induced in the women being subthreshold (potentially from the smaller muscle mass) or due to other sex-based differences is unclear. In Lewis rats, where there is a similar hindlimb to body-mass ratio between sexes, sex differences in myocardial protection from remote ischemic preconditioning are abolished, arguing for the influence of muscle mass in RIC outcomes (70). In any case, given the more severe illness and higher mortality rate seen in men, coupled with potential increased responsiveness to ischemic conditioning, it is reasonable to speculate that a greater impact of any ischemia-based therapy would be seen in males.

## LIMITATIONS

The seminal discovery in humans by Loukogeorgakis et al. (37) that RIC can be successfully applied during a pathological stressor significantly enhances the clinical utility of this adjunct therapy, which has since shown promise in patients with ST-segment elevated myocardial infarction (71). Although protection can be mediated during or immediately postinsult, there are temporal limits that confine the application of ischemic per- and postconditioning. For many patients with COVID-19, symptoms are mild and recovery is largely uneventful. More severe cases may not develop overt symptoms and present for treatment until well after infection and only after a prolonged period of lung injury has been allowed to occur. As a result, RIC may face the significant challenge of trying to reverse injury that is established and beyond rescue. Nevertheless, current reports describe a worsening of condition during hospitalization in severe patients (3–5), and thus application of ischemic perconditioning upon intake, before further life-threatening organ injury ensues, may be warranted.

Most experimental models have tested the benefits of ischemic conditioning under focused disease conditions, absent of comorbidities. The human condition is typically much more complex and this challenge is seen in patients with COVID-19. The most severe clinical conditions of SARS-CoV-2 infection have a higher likelihood of manifesting in older patients, often with a number of concomitant health challenges (3–6, 14). Studies show that comorbidities and other complicating factors, such as aging, can diminish the effectiveness of ischemic conditioning (27). The weakened effectiveness of ischemic conditioning under circumstances that are common among the most severely ill patients with COVID-19 may serve as a significant obstacle to the clinical exploitation of the phenomenon. In addition, given the absence of studies assessing RIC in lung infection, we resorted to interpreting animal and human models of lung IR given the focal lung insult and inflammatory cascades induced by these procedures, bringing similarities to pathophysiologic mechanisms of COVID-19. Noteworthy, however, many lung IR models involve surgical procedures requiring mechanical ventilation. Alongside IR-induced lung injury, lung injury related to different mechanisms associated with mechanical ventilation, such as barotrauma, volutrauma, and atelectrauma, are also relevant (72). We speculate that the protective effects of RIC likely combat the IR-mediated lung injury mechanisms, but given the presence of alternative mechanisms of injury in these models, generalizability of these results should be cautioned.

The benefits of pre-, per-, and post-conditioning have been demonstrated in a number of animal models subjected to different stressors across a multitude of organ systems. Despite clear evidence in nonhuman animals, the clinical translation of RIC has proven problematic and is a clear limitation of its potential. The RIC-STEMI randomized clinical trial of 448 patients diagnosed with ST-elevated myocardial infarction (STEMI) showed that RIC decreased cardiac mortality and hospitalization for heart failure (73). By contrast, the CONDI-2/ERIC-PPCI clinical trial found no significant benefits on cardiac death or hospitalization at 12 mo (74). Differences including the duration of the ischemia-reperfusion cycles have been cited as potential sources for these discrepancies. In fact, in a review of the translational obstacles that face ischemic conditioning, it was noted that there have been no phase II dosing and timing trials to determine effective treatment protocols (75). The mitigating effects of propofol on patients have been cited in a number of negative trials and reviews, and positive trials have been criticized for their relatively small sample size, underpowered analysis, and risk for type 1 errors. Until these fundamental issues are addressed and sufficiently powered randomized clinical trials are conducted, the potential clinical translation of RIC will likely remain unrealized.

## CONCLUSIONS

Pneumonia-induced ARDS and subsequent respiratory failure remains the leading cause of death in patients severely impacted by COVID-19, requiring novel adjunct therapeutic approaches. RIC of the limbs has shown potential in recent years to protect the lungs (and other organs) against pathological conditions similar to that observed in COVID-19 (Fig. 1*B*). Ischemic preconditioning has been proven safe for use in a variety of clinical cohorts (63, 64). Though development of novel antiviral treatments and vaccines hold promise in control and prevention of early disease, it is noteworthy that in severe cases of COVID-19, addressing “run-away” inflammatory cascades are likely more relevant for improvement of clinical outcomes, given that viral loads may decrease in severe, end-stage coronavirus cases (68), unlike the cytokine storm (3, 4). Mitigating the inflammatory risk after the control of the infection will likely prove critical in determining the ultimate patient outcome. The present mini-review focused on drawing parallels between RIC and clinical targets of COVID-19. The testing of RIC as an easily administered and accessible countermeasure for COVID-19, with little to no risk for patients, represents a viable hypothesis which warrants investigation.

## GRANTS

This work was supported by a Natural Science and Engineering Research Council of Canada (NSERC) Canada Graduate Scholarship and a Canadian Institutes of Health Research (CIHR) Fellowship (to A.V.I.); NSERC Discovery Award No. 04732 and CIHR Project Grant 437970 (to W.G.P.); NSERC Discovery Grant No. 04287, Canada Foundation for Innovation, and an Ontario Early Research Award (to P.J.M.).

## DISCLOSURES

No conflicts of interest, financial or otherwise, are declared by the authors.

## AUTHOR CONTRIBUTIONS

A.V.I. prepared figures; A.V.I., P.J.M., and W.G.P. drafted manuscript; A.V.I., P.J.M., and W.G.P. edited and revised manuscript; A.V.I., P.J.M., and W.G.P. approved final version of manuscript.
